# Elixhauser comorbidity method in predicting death of Spanish inpatients with asplenia and pneumococcal pneumonia

**DOI:** 10.1186/s12879-024-09517-4

**Published:** 2024-06-20

**Authors:** Enrique Gea-Izquierdo, Rossana Ruiz-Urbaez, Valentín Hernández-Barrera, Michael Stich, Ángel Gil-de-Miguel

**Affiliations:** 1 Faculty of Medicine, Pontifical Catholic University of Ecuador, Quito, Ecuador; 2 Department of Medical Specialties and Public Health, Rey Juan Carlos University, Madrid, Spain; 3 María Zambrano Program, European Union, Madrid, Spain; 4 Internal Medicine Service, Eugenio Espejo Hospital, Quito, Ecuador; 5 Department of Applied Mathematics, Materials Science and Engineering, and Electronic Technology, Rey Juan Carlos University, Madrid, Spain; 6 CIBER of Respiratory Diseases (CIBERES), Instituto de Salud Carlos III, Madrid, Spain

**Keywords:** Asplenia, Pneumococcal disease, Elixhauser Comorbidity Index, Spain

## Abstract

**Background:**

Pneumococcal pneumonia (PP) is a serious infection caused by *Streptococcus pneumoniae* (pneumococcus), with a wide spectrum of clinical manifestations. The aim of this study was to analyze the comorbidity factors that influenced the mortality in patients with asplenia according to PP.

**Methods:**

Discharge reports from the Spanish Minimum Basic Data Set (MBDS) was used to retrospectively analyze patients with asplenia and PP, from 1997 to 2021. Elixhauser Comorbidity Index (ECI) was calculated to predict in-hospital mortality (IHM).

**Results:**

97,922 patients with asplenia were included and 381 cases of PP were identified. The average age for men was 63.87 years and for women 65.99 years. In all years, ECI was larger for splenectomized than for non-splenectomized patients, with men having a higher mean ECI than women. An association was found between risk factors ECI, splenectomy, age group, sex, pneumococcal pneumonia, and increased mortality (OR = 0.98; 95% CI: 0.97–0.99; *p* < 0.001). The IHM increased steadily with the number of comorbidities and index scores in 1997–2021.

**Conclusions:**

Asplenia remain a relevant cause of hospitalization in Spain. Comorbidities reflected a great impact in patients with asplenia and PP, which would mean higher risk of mortality.

## Background

Pneumococcal pneumonia (PP) is commonly encountered in elderly populations who are increasingly multimorbid due to advances in life expectancy and in medical care [1]. Comorbidity is defined as the coexistence of multiple conditions related with an index medical condition at an individual patient level. It is usually measured by the sum of the number of diseases present in a patient, differentiating the main diagnosis from the secondary ones. Sometimes the outcomes of interest are often influenced by concurrent or preexisting comorbidities and therefore comorbidity may be defined as the total burden of illnesses unrelated to the principal diagnosis [2]. PP rarely occurs in isolation, with patients often having more than one additional comorbid condition. The burden of comorbidity is important in patients with asplenia [3, 4].

There is increasing interest in using measures of comorbidity burden in developing risk-stratification tools in patients with PP. There are several tools to measure comorbidity, some of the most frequently used ones are the Charlson Comorbidity Index (CCI) and Elixhauser Comorbidity Index (ECI). Both are well-validated measures of comorbid burden and can be applied for risk assessment in patients with asplenia and PP. The first consists of 19 predefined comorbidities to which a value is assigned and the second is a list of 30 conditions that allow the risk of death to be calculated without giving any weight to the type of pathology. These comorbidity indices have been widely used in studies using administrative data to control for the overall burden of comorbidities [5]. Nevertheless, the ECI has been developed exclusively for use with administrative data [6], whereas other indices have been developed in different contexts but adapted for this use, such as the CCI [7, 8]. The appropriate selection of a comorbidity index for use with administrative data should consider the type of data available, the study population, and the specific outcome of interest in the study.

Complicated pneumonia is that which presents pleural effusion, lung abscess or parenchymal necrosis. It has been seen that in recent years the proportion of complicated pneumonias has increased, mainly empyema and, to a lesser extent, necrotizing forms. Normally, the most common complication of all of them is pleural effusion, with *Streptococcus pneumoniae* (SP) being the main etiological agent, although the serotypes have varied over time with the introduction of the different vaccines against this microorganism. Necrotizing pneumonia and lung abscess are fewer common complications [9]. Pneumococcus is the current etiological agent that most frequently conditions the suppurative complications of pneumonia. Since the introduction of the vaccine against pneumococcus, it is known that certain serotypes tend more easily to evolve towards lung abscess and necrotizing pneumonia. The incidence increase of the necrotizing pneumonia in the last two decades has been linked with a raise in the production of interleukin 8 (IL-8) in certain serotypes of pneumococci (serotype 19-A and 3) and with a higher number of them in the pleural cavity [10, 11].

The objective of the study aims to analyze the comorbidity factors that influence the mortality in patients with asplenia due to PP.

## Methods

### Study design

A cross-sectional and retrospective study was carried out. The hospital discharge report was obtained from the Government of Spain. Patients with asplenia (splenectomized/non-splenectomized) were extracted from the Minimum Basic Data Set (MBDS) of the Spanish Ministry of Health.

### Data source and study population

All hospital discharges with PP in Spain from the year 1997 to 2021 were extracted from the MBDS, which is the largest publicly available inpatient healthcare database in Spain. The MBDS database includes diagnosis and procedure codes, which were used to identify relevant conditions and comorbidities. The database includes a stratified sample of hospitalizations from 98% participating hospitals to improve national estimates [12]. Weights were recorded for each discharge record, which was applied in the analysis to obtain national estimates.

The study period was from January 1997 to December 2021. All patients aged 40 years or older with a principal diagnosis of asplenia were included and identified by International Classification of Diseases (ICD), Ninth Edition, Clinical Modification (ICD-9-CM) (splenectomy, 41.5, 41.43; no splenectomy, 865, 759.0, 289.59) and 10th Revision, ICD-10-ES (splenectomy, 07TP0ZZ; no splenectomy, Q89.01, D73.3, D73.4, D73.5, D73.89, Q89.09). The definition of SP used according to the ICD-9-CM and ICD-10-ES codes was 481 and J13, respectively.

The distribution of the population was analyzed by calculating the global dependency indices. The primary outcome in this study was in-hospital mortality (IHM), defined as all-cause death during the period of hospitalization. Algorithms were developed to calculate the ECI adapted for use with the ICD9 from secondary diagnoses from the discharge data of each patient.

### Hospitalization and demographic data

Hospital data contains all the relevant diagnoses during an episode of care as an inpatient. For each diagnosis recorded in the hospital data, a corresponding variable called diagnosis type is used to identify whether the diagnosis is considered a comorbidity (pre-existing condition) or a complication (a condition arising during the hospital stay).

Different clinical variables and sociodemographic variables were obtained. The study variables were health diagnosis (asplenia), admission day, age, sex, smoker, malignant pleural effusion, non-specific pleural effusion, lung abscess, mediastinal abscess, empyema with fistula, empyema without fistula, and pneumococcal pneumonia. In addition to clinically relevant comorbidities included from baseline patient characteristics, Elixhauser comorbidities were considered; hospital characteristics, and inpatient outcomes such as discharge disposition and hospital length of stay also were collected.

Baseline characteristics were retrospectively collected and Elixhauser comorbidity score (ECS) was calculated. Baseline medical history and comorbidities were administered. The ECI was calculated according to the point system from van Walraven [13]. The van Walraven modification simplifies the ECS calculation by eliminating some variables.

The MBDS data contain only one diagnosis code per record. The ECI is originally designed to work with hospital data only. Recognizing its utility to answer valuable clinical questions, the MBDS has been used to analyze data associated with a wide range of diagnoses and procedures.

### Statistical analysis

Data were statistically managed using the Stata software version 16.1. Results of the descriptive statistics were expressed in percentage, mean and standard deviation. Variables indicating the presence or absence of each Elixhauser comorbidity were created, and their associations with mortality were assessed in bivariate analysis. To study the possible relationship between qualitative variables, the Pearson’s chi-squared test (or Fisher’s exact test in cases where that was necessary) were used. To study the relationship between quantitative variables the Student t-test and ANOVA were implemented. Multivariable analysis mortality model was done using non-conditional logistic regression analysis, analyzing the requirements of the confounding variables, and excluding the collinearity. In all tests, the significance level used was *p* < 0.001.

30 comorbidity binary variables proposed by Elixhauser et al. [6] were used. For a current list of the categories in the ECI used in the study the ICD-9-CM and ICD-10-ES diagnosis codes from both hospital data and medical services data were used. In addition to this index, an exhaustive analysis of specific comorbidities was conducted. To carry out the analysis of the different comorbidities potentially associated with PP, the ECI was applied. A specificity was required to distinguish between diagnoses that should/should not be included in the index and to be able to properly identify and place codes into the appropriate category [14].

In addition, the weighted ECS, developed by van Walraven et al., was computed and further stratified into groups (40–49, 50–59, 60–69, 70–79 and > 80 years, respectively). The Elixhauser weights assigned to each comorbidity range from < 0 to *≥* 5. Comorbidity scores then can be calculated for each patient by summing the individual weights of all comorbidities.

Elixhauser risk index in all Spanish patients with asplenia and PP was analyzed. According to the scores obtained, different risk groups of mortality were determined. Crude mortality rates and group-specific age and sex were calculated by dividing the number of deaths by PP by the number of PP cases in each subgroup and were expressed as percentages. Only cases of PP were studied in patients 40 years of age or older. Multivariable logistic regression analyses were performed to assess the contributions of the individual Elixhauser comorbidities to predicted IHM. A comorbidity risk adjustment model regarding its ability to predict IHM was assessed.

### Ethical statement

Since de-identified data were used in this study, patient consent to review their medical records was not required.

## Results

**Fig. 1:**
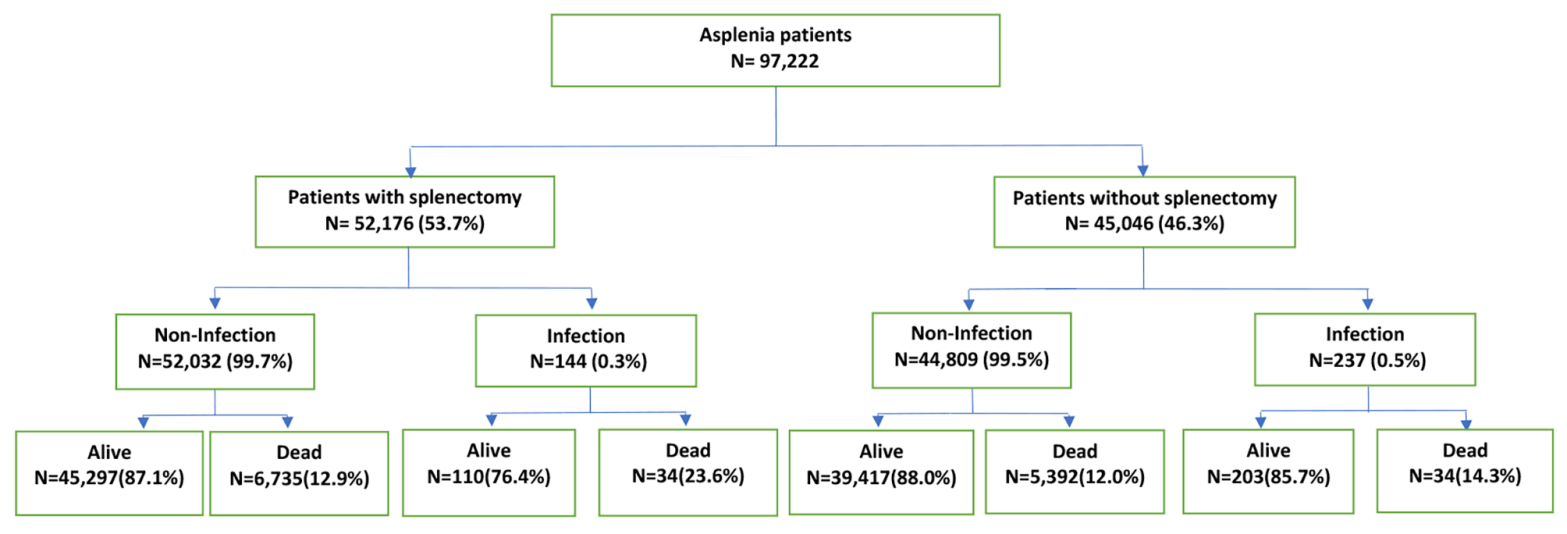
Flowchart of patients with asplenia

Discharges from the period 1997–2021 were analyzed. After application of the algorithms, 97,922 asplenia cases were identified (52,176 splenectomized and 45,046 non-splenectomized) from the Spanish database (Fig. 1). The average age for men was 63.87 years and for women 65.99 years. From 1997, 381 cases of PP were identified, more men than women were admitted (57,522/39,700). The average age of admission has increased progressively in both sexes, although that of women is older, and those admitted have comorbidities with PP at the time of admission.

The results of the comparison of comorbidities in patients with asplenia and PP and non-PP were analyzed. The prevalence differences were moderate and in no case statistically significant (except non-specific pleural effusion; *p*-value = 0,011). When comparing the evolution of comorbidity index at the time of patient admission with PP, in all years the average was greater in splenectomized than in the non-splenectomized. The differences were statistically significant in most cases and that the average values of these indices increased constantly in both patients with asplenia.

Considering comorbidities, ECI-van Walraven, mean + SD were determined (Table 1). The distribution of the Elixhauser index by age groups was observed, evidencing significant differences between them. The group 70–79 years old had higher values than the rest. Male-female proportion and the total number of PP cases in the period studied with each specific comorbidity which is part of the Elixhauser index was calculated. Table 2 shows how the Elixhauser index is distributed between men and women. There were significant differences between sexes, with men having a higher mean Elixhauser index than women. According to Elixhauser Comorbidity Index (*≥* 5), the number of patients (%) increased 18.85% from 40 to 49 to 70–79 years. According to the bivariate analysis, a ECI was found to be significantly correlated with PP (*p* < 0.001).

**Table 1:** Relationships between the elixhauser comorbidity index-van walraven (mean + SD) and clinical parameters

		Total	Elixhauser Comorbidity Index-van Walraven (mean ± SD)
Splenectomy	No	45,046	8.01 ± 8.34
	Yes	52,176	7.55 ± 7.49
Age group	40–49 years	15,191	4.77 ± 6.97
	50–59 years	19,505	7.01 ± 7.79
	60–69 years	23,893	8.13 ± 7.85
	70–79 years	25,591	8.83 ± 7.89
	*≥* 80 years	13,042	9.61 ± 8.11
Sex	Male	57,522	7.83 ± 7.93
	Female	39,700	7.66 ± 7.86
Smoker	No	86,580	7.79 ± 7.94
	Yes	10,642	7.5 ± 7.6
Malignant pleural effusion	No	96,993	7.74 ± 7.89
	Yes	229	17.29 ± 7.5
Non-specific pleural effusion	No	92,122	7.73 ± 7.9
	Yes	5100	8.41 ± 7.83
Lung abscess	No	97,075	7.76 ± 7.9
	Yes	147	7.63 ± 7.9
Mediastinal abscess	No	97,205	7.76 ± 7.9
	Yes	17	3.29 ± 3.44
Empyema with fistula	No	97,112	7.76 ± 7.9
	Yes	110	6.71 ± 8.11
Empyema without fistula	No	96,837	7.76 ± 7.9
	Yes	385	7.74 ± 7.65
Pneumococcal pneumonia	No	96,841	7.76 ± 7.9
	Yes	381	7.13 ± 7.61
Death	No	85,027	7.3 ± 7.65
	Yes	12,195	11 ± 8.82
Total		97,222	7.76 ± 7.9

**Table 2:** Relationships between the elixhauser comorbidity index-van walraven (< 0 vs. ≥ 5) and clinical parameters

		Elixhauser Comorbidity Index-van Walraven				
		< 0	0	1–4	*≥* 5	*p*-value
Splenectomy; *n* (%)	No	2524(58.34)	11,327(48.97)	5549(30.66)	25,646(49.64)	0.000
	Yes	1802(41.66)	11,805(51.03)	12,552(69.34)	26,017(50.36)	
Age; mean (SD)		60.88(13.3)	59.74(12.98)	65.45(12.25)	67.05(12.43)	0.000
Age group; *n* (%)	40–49 years	1122(25.94)	6267(27.09)	2356(13.02)	5446(10.54)	0.000
	50–59 years	955(22.08)	5830(25.2)	3461(19.12)	9259(17.92)	
	60–69 years	965(22.31)	5179(22.39)	4693(25.93)	13,056(25.27)	
	70–79 years	902(20.85)	4087(17.67)	5366(29.64)	15,236(29.49)	
	*≥* 80 years	382(8.83)	1769(7.65)	2225(12.29)	8666(16.77)	
Sex; *n* (%)	Male	1959(45.28)	14,283(61.75)	10,608(58.6)	30,672(59.37)	0.000
	Female	2367(54.72)	8849(38.25)	7493(41.4)	20,991(40.63)	
Smoker; *n* (%)	No	3756(86.82)	20,803(89.93)	15,997(88.38)	46,024(89.09)	0.000
	Yes	570(13.18)	2329(10.07)	2104(11.62)	5639(10.91)	
Malignant pleural effusion; *n* (%)	No	4324(99.95)	23,130(99.99)	18,092(99.95)	51,447(99.58)	0.000
	Yes	2(0.05)	2(0.01)	9(0.05)	216(0.42)	
Non-specific pleural effusion; *n* (%)	No	4128(95.42)	22,076(95.43)	17,237(95.23)	48,681(94.23)	0.000
	Yes	198(4.58)	1056(4.57)	864(4.77)	2982(5.77)	
Lung abscess; *n* (%)	No	4319(99.84)	23,102(99.87)	18,070(99.83)	51,584(99.85)	0.745
	Yes	7(0.16)	30(0.13)	31(0.17)	79(0.15)	
Mediastinal abscess; *n* (%)	No	4326(100)	23,125(99.97)	18,097(99.98)	51,657(99.99)	0.245
	Yes	0(0)	7(0.03)	4(0.02)	6(0.01)	
Empyema with fistula; *n* (%)	No	4322(99.91)	23,096(99.84)	18,077(99.87)	51,617(99.91)	0.068
	Yes	4(0.09)	36(0.16)	24(0.13)	46(0.09)	
Empyema without fistula; *n* (%)	No	4310(99.63)	23,045(99.62)	18,028(99.6)	51,454(99.6)	0.935
	Yes	16(0.37)	87(0.38)	73(0.4)	209(0.4)	
Pneumococcal pneumonia; *n* (%)	No	4309(99.61)	23,046(99.63)	18,022(99.56)	51,464(99.61)	0.748
	Yes	17(0.39)	86(0.37)	79(0.44)	199(0.39)	
Death; *n* (%)	No	4109(94.98)	21,434(92.66)	16,384(90.51)	43,100(83.43)	0.000
	Yes	217(5.02)	1698(7.34)	1717(9.49)	8563(16.57)	
Hospital stay; median (IQR)		11(15)	11(14)	13(17)	13(18)	0.000

Significant differences have also been found in the ANOVA analysis between the age groups. Table 3 shows the distribution of the ECI and comorbidities without or with PP, with significant differences in them. In this table is shown that ECI (*≥* 5), age (50–59 years) and empyema with fistula were significant risk factors for PP. Smoker comorbidity was shown as not risky (without/with PP), among others, understood as that there are patients who feel better and continue smoking and those who feel worse have stopped smoking (sometimes occurs with MBDS). The contrary happens in empyema with fistula. However, non-specific pleural effusion was a risk factor for patients without PP (*p* < 0.001) and the opposite with PP. The PP prevalence was 4.25 times higher between 1997 and 2021 (0.85 and 0.20 respectively). The PP prevalence was 0.28 vs. 0.53 in the splenectomy group and non-splenectomy group, respectively. After adjusted by sex and age, the OR of PP was 1.87 times higher in the non-splenectomy group than in the other.

**Table 3:** Risk factors for IHM in patients without/with PP

	Without PP	*p*-value	With PP	*p*-value	Total	*p*-value
Elixhauser Comorbidity Index-van Walraven						
<0	1 (reference)		NA		1 (reference)	
0	1.45(1.25–1.67)	0.000	1		1.45(1.26–1.68)	0.000
1–4	1.57(1.35–1.82)	0.000	1.33(0.48–3.72)	0.002	1.58(1.36–1.83)	0.000
*≥*5	2.95(2.57–3.4)	0.000	3.72(1.61–8.59)	0.009	2.97(2.59–3.42)	0.000
Splenectomy	1.24(1.19–1.29)	0.000	1.9(1.07–3.36)	0.029	1.25(1.2–1.3)	0.000
Age						
40–49 years	1 (reference)		1 (reference)		1 (reference)	
50–59 years	1.29(1.18–1.4)	0.000	2.91(0.93–9.16)	0.067	1.29(1.19–1.41)	0.000
60–69 years	1.75(1.62–1.89)	0.000	1.36(0.42–4.43)	0.613	1.75(1.62–1.89)	0.000
70–79 years	2.45(2.26–2.64)	0.000	2.41(0.82–7.14)	0.111	2.45(2.26–2.64)	0.000
*≥*80 years	3.81(3.51–4.13)	0.000	1.36(0.41–4.59)	0.616	3.79(3.5–4.11)	0.000
Sex						
Male	1.35(1.29–1.4)	0.000	1.11(0.6–2.04)	0.747	1.34(1.29–1.4)	0.000
Female	1 (reference)		1 (reference)		1 (reference)	
Smoker	0.71(0.66–0.76)	0.000	0.21(0.06–0.74)	0.015	0.71(0.66–0.76)	0.000
Malignant pleural effusion	2.49(1.86–3.33)		NA		2.49(1.86–3.33)	0.000
Non-specific pleural effusion	1.34(1.24–1.45)	0.000	0.38(0.11–1.32)	0.128	1.33(1.23–1.44)	0.000
Lung abscess	2.09(1.39–3.13)	0.000	NA		2.23(1.5–3.31)	0.000
Mediastinal abscess	0.38(0.05–2.96)	0.360	NA		0.38(0.05–2.95)	0.357
Empyema with fistula	4.08(2.67–6.23)	0.000	8.41(0.21-336.82)	0.258	4.09(2.69–6.21)	0.000
Empyema without fistula	1.54(1.18–2.02)	0.002	NA		1.48(1.13–1.94)	0.004
Pneumococcal pneumonia	NA		NA		1.48(1.13–1.93)	0.005

According to the ECI, the comorbidities that most predominated in patients in whom the first diagnosis was PP were: hypertension (21.26%), chronic pulmonary disease (20.21%), solid tumor without metastasis (18.37%), cardiac arrhythmias (14.96%), and liver disease (14.44%) (Table 4). There was evidence of epidemiological differences between PP in patients with asplenia, including older age, and higher age adjusted ECI scores, rising up to 9.61 (*≥* 80 years) at the Elixhauser Comorbidity Index.

**Table 4:** Comorbidities in patients with PP diagnosis

Comorbidities	%
Hypertension, uncomplicated	21.26
Chronic pulmonary disease	20.21
Solid tumor without metastasis	18.37
Cardiac arrhythmias	14.96
Liver disease	14.44
Diabetes, uncomplicated	11.29
Alcohol abuse	11.02
Congestive heart failure	8.92
Coagulopathy	8.92
Metastatic cancer	8.40
Renal failure	7.87
Fluid and electrolyte disorders	7.35
Hypertension, complicated	6.04
Depression	5.77
Peripheral vascular disorders	5.51
Weight loss	5.51
Valvular disease	4.99
Other neurological disorders	4.99
Lymphoma	4.99
Pulmonary circulation disorders	4.72
Rheumatoid arthritis/Collagen vascular diseases	4.72
Obesity	3.94
Deficiency anemia	3.41
Diabetes, complicated	2.89
Hypothyroidism	2.62
AIDS/HIV	2.10
Drug abuse	2.10
Blood loss anemia	1.57
Peptic ulcer disease excluding bleeding	1.05
Psychoses	1.05
Paralysis	0.52

**Fig. 2:**
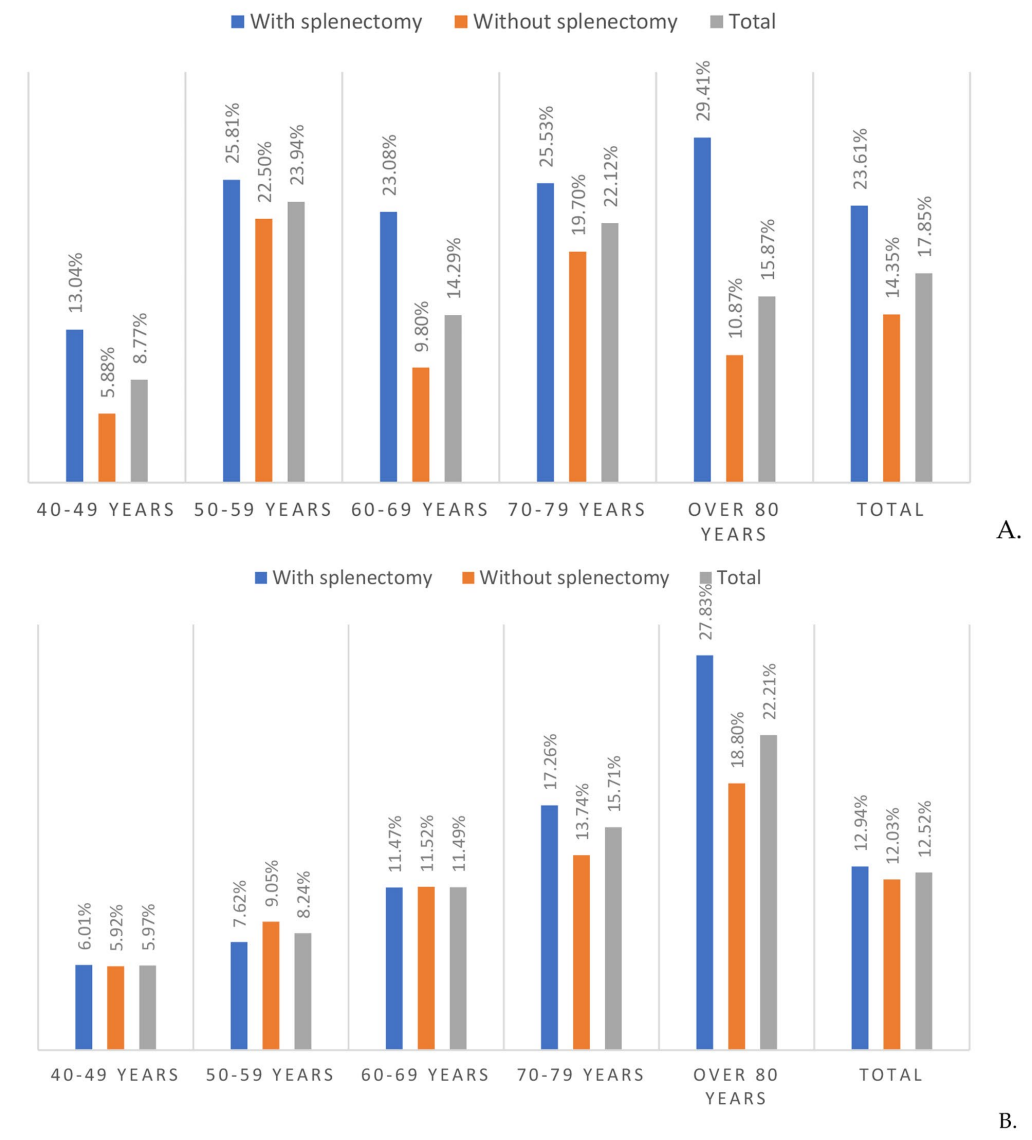
IHM of the patients with asplenia by age group in Spain, from 1997 to 2021. (**A**) With pneumococcal pneumonia. (**B**) Without pneumococcal pneumonia

To assess the impact of comorbidities on mortality by PP a multivariable model was developed. IHM of the patients with asplenia (with and without splenectomy) and pneumococcal pneumonia (without and with) by age group is shown in Fig. 2. An association was found between risk factors ECI, splenectomy, age group, sex, pneumococcal pneumonia, and increased mortality in patients admitted during the period 1997–2021 (OR = 0.98; 95% CI: 0.97–0.99; *p* < 0.001) (Table 3). Likewise, it analyzed whether there was an association between PP and malignant pleural effusion, lung abscess, and empyema with fistula and a possible effect was found risk during the same period (OR = 2.49, 95% CI:1.86–3.33, *p* < 0.001; OR = 2.23, 95% CI:1.5–3.31, *p* < 0.001; OR = 4.09, 95% CI:2.69–6.21, *p* < 0.001; respectively). The IHM increased steadily with the number of comorbidities and index scores in 1997–2021.

## Discussion

During the period 1997–2021, there was a considerable increase in comorbidities of patients with asplenia admitted with PP. This increase was detected with the ECI. Although some studies on mortality in Spain have used other indices, there is none that have used the Elixhauser index in patients with asplenia and PP. Therefore, this is the first study in Spain that uses this index in the assessment of mortality due to PP.

The comparative analysis of the prevalence of comorbidities indicates that these are a relative number, with differences statistically significant. However, when comparing the prevalence of different diseases, it is observed that some differences could be a consequence of underdiagnosis of the diseases, although it should be verified with a more exhaustive analysis.

In 1997–2021, 64.31% of the Spanish population with asplenia was 60 or older of age and people aged 80 or older were 13.41% of the entire population. The ECI has a greater correlation with the adjusted morbidity group. Differences have been identified between the calculated index and age group. The distribution of the population with asplenia is mostly male, and over 60 years. There is an increase in the index in 80 year or more. The ECI has a similar average score for men and women. It should be noted that men have more diagnoses than women and, in relation to this, greater comorbidity. The increase in the male population may be a consequence of immigration in Spain that already affects the total population. Differences between sexes are also observed when comparing the number of diagnoses, with men having the highest average. Likewise, it would be advisable to differentiate between the origin of the population since there is many foreign populations.

The most relevant results of the present study are shown by the ECI, detected those patients at risk of death. The simplicity of the index and their obtaining in epidemiological surveillance is a useful tool to carry out audits of operative results. For patients of higher risk, the main cause of death was pneumonia (ICD-9-CM: 481, ICD-10-ES: J13). In the study, the ECI clearly detected high-risk patients (53.14%) and determined a group of low-risk patients (4.45%). The factors that differentiated these groups of patients were the PP complications (0.39%). Fluid and electrolyte disorders, coagulopathy, and congestive heart failure were the major factors influencing mortality from the variables in the ECI. These results suggest that the ECI functions as a prognostic factor in cases of PP.

The increase in the values of the comorbidity index did not occur only among splenectomized patients, but also among non-splenectomized. This allows us to reflect on the impact that comorbidities could have in the future in patients with asplenia and PP, which would mean higher healthcare costs and a higher risk of mortality [15]. The ECI was a major factor influencing mortality. Some comorbidities (deficiency anemia, hypothyroidism, drug abuse, obesity) were associated with decreased odds of inpatient mortality but it is counterintuitive that these comorbidities would protect against inpatient death. One possibility is that they are more common in patients with less overall infirmity.

Surgical mortality and morbidity are important parameters of in-hospital quality of care [16, 17]. ECS was considered in predicting mortality in patients with asplenia and in predicting inpatient death after pneumococcal infections. The Elixhauser comorbidity risk performed numerically good in predicting IHM after PP. Most of the Elixhauser comorbidities influenced IHM. The risk factors for PP in patients with asplenia (OR) was significant for all comorbidities (Table 3). Comorbidities most strongly associated with IHM included ECS ≥ 5 (OR: 2.97, 95% CI: 2.59–3.42), empyema with fistula (OR: 4.09, 95% CI: 2.69–6.21) and malignant pleural effusion (OR: 2.49, 95% CI: 1.86–3.33). However, only smoker and mediastinal abscess comorbidities were significantly associated with a decreased risk of IHM. The points assigned to each comorbidity group ranged from − 7 (drug abuse) to 3.55 (metastatic cancer). The range of possible ECS was < 0 to ≥ 5. Table 2 shows the relationships between the Elixhauser Comorbidity Index-van Walraven and clinical parameters. Each outcome was significantly associated with the ECS. The overall value of each outcome measure increased as the ECS increased. IHM increased as the ECS increased with the burden of illness. Therefore, it is estimated that although the score did not accurately predict risk of IHM, it can be used to describe and adjust for comorbidity in patients with asplenia and PP.

Despite the increase in age and the greater number of comorbidities, mortality continues to decline, indicating the Spanish healthcare standards of the services involved in the care of patients with asplenia. Therefore, the Elixhauser risk score should be used in clinical decision making when mortality assessment is required when selecting patients with PP.

This study has some limitations. The data that have been used are those contained in the MBDS and have not been supplemented with additional patient data. The analysis is limited to mortality during the hospital stay, since no data is available on the patient’s mid to late-term evolution. The difficulty of defining the basic cause before a patient who is admitted with many simultaneous illnesses and without available background can influence the quality of the diagnoses that are recorded in the clinical history and, therefore, in the MBDS. Another limitation is the potential underreporting of information; and the lack of clinical, lifestyle, and demographic data, because of the “observational nature”, which can introduce biases. Other biases are channeling bias, as well as confounding by indication, although may have been minimized by the adjustment in the statistical analyses. Comorbidity index provides only a limited ability to control for confounding, acknowledging nonetheless its usefulness because of ease of use and time and resources savings. Additionally, the possibility of errors in coding of the diagnoses and procedures cannot be avoided. The results must be observed with caution because of the differences that may have occurred until 2009 may differ from this year due to the introduction of the POA indicator (present on admission). In the coming years it will be interesting to validate these findings, since all MBDS diagnoses will be accompanied by the POA indicator [18]. Finally, the MBDS enabled only ascertainment of inpatient outcomes, and thus post discharge complications and readmissions are not considered. It could be estimated the possibility of bacterial superinfection in patients with asplenia and PP during the course, a factor that could contribute significantly to higher mortality.

An advantage is that all MBDS variables are collected in 98% hospitals in Spain, so the calculation of this index and the predictive model of mortality due to PP could be done systematically in all health centers and could be used for the analysis of the quality of care.

ECI provided clinically relevant insight for estimating nonroutine discharge. This finding suggests that the index is a valid prediction tool for healthcare resource use risk adjustment, and researchers should choose between them based more on their availability and comfort with the method [19]. Future research assessing and comparing the performance in predicting long-term pneumococcus infection would be of value. Other alternatives could be implemented in selecting a comorbidity index in other related studies, including when administrative data are available on both diagnosis and medication information. Inpatient mortality may be evaluated differently depending on the comorbidity risk adjustment model selected. Analyses of risk-adjusted mortality rates should adjust mortality rates only for baseline comorbid diseases. Future research assessing and comparing the performance of the Elixhauser measures in predicting long-term outcomes would be of value in patients with asplenia and PP.

## Conclusions

Asplenia remain a relevant cause of hospitalization in Spain. PP shows a significant IHM in patients with asplenia. ECI is larger for splenectomized than for non-splenectomized patients. In medium-older age, the IHM increases steadily with the number of comorbidities and the ECI during the 25 years of this survey. Elixhauser index is a fine estimative tool in predicting outcomes in patients with asplenia and PP.

## Data Availability

The datasets analyzed in the current study are publicly available in the Hospital Discharge Records in the Spanish National Health System (MBDS) repository at https://www.mscbs.gob.es/en/estadEstudios/estadisticas/cmbdhome.htm (accessed on 26 January 2024). The information contained in this repository can be accessed without the need for any administrative permissions.

## Abbreviations

CCI: Charlson Comorbidity Index
ECI: Elixhauser Comorbidity Index
ICD: International Classification of Diseases
ICD-10-ES: International Classification of Diseases 10th Revision
ICD-9-CM: International Classification of Diseases 9th Revision
IHM: In-hospital mortality
MBDS: Minimum Basic Data Set
OR: Odds ratio
PP: Pneumococcal pneumonia
SP: *Streptococcus pneumoniae*
